# Absorbable Electrospun Poly-4-hydroxybutyrate Scaffolds as a Potential Solution for Pelvic Organ Prolapse Surgery

**DOI:** 10.1021/acsabm.2c00691

**Published:** 2022-10-31

**Authors:** Kim Verhorstert, Aksel Gudde, Carmen Weitsz, Deon Bezuidenhout, Jan-Paul Roovers, Zeliha Guler

**Affiliations:** † Department of Obstetrics and Gynecology, Amsterdam UMC, University of Amsterdam, Meibergdreef 9, 1105 AZAmsterdam, The Netherlands; ‡ Amsterdam Reproduction and Development Research Institute, Meibergdreef 9, 1105 AZAmsterdam, The Netherlands; § Cardiovascular Research Unit, Department of Surgery, University of Cape Town, 203 Chris Barnard Building, Anzio Road, Observatory7925Cape Town, South Africa

**Keywords:** pelvic organ prolapse (POP), electrospinning, scaffold, implant, mesh, estradiol, absorbable, poly-4-hydroxybutyrate (P4HB)

## Abstract

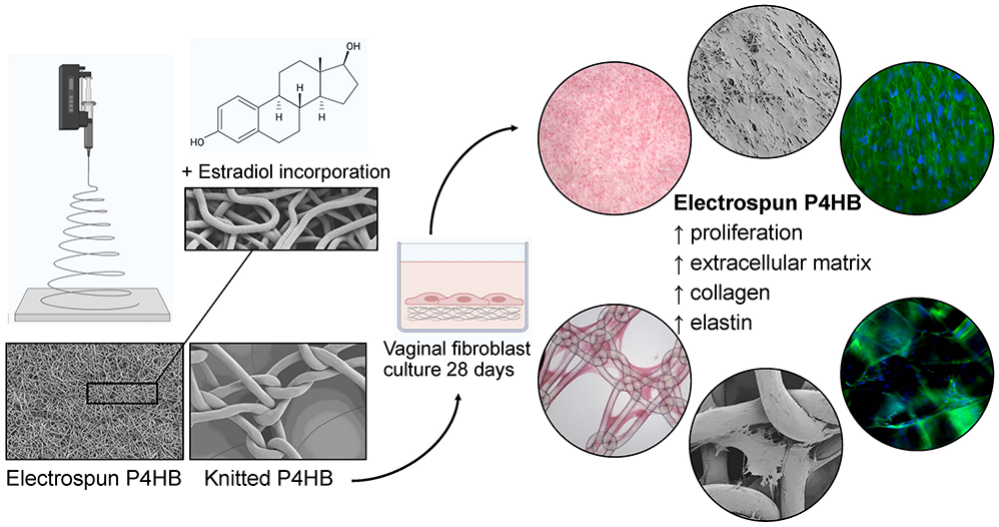

Women with pelvic organ prolapse (POP) have bothersome complaints that significantly affect their quality of life. While native tissue repair is associated with high recurrence rates, polypropylene knitted implants have caused specific implant-related adverse events that have detrimental, often irreversible, effects. We hypothesize that surgical outcome can be improved with a tissue-engineered solution using an absorbable implant that mimics the natural extracellular matrix (ECM) structure, releases estrogen, and activates collagen metabolism by fibroblasts as the main regulators of wound healing. To this aim, we produced electrospun poly-4-hydroxybutyrate (P4HB) scaffolds and biofunctionalized them with estradiol (E2). The cell–implant interactions relevant for POP repair were assessed by seeding primary POP vaginal fibroblasts isolated from patients on electrospun P4HB scaffolds with 1%, 2%, or 5% E2 and without E2. To test our hypothesis on whether ECM mimicking structures should improve regeneration, electrospun P4HB was compared to knitted P4HB implants. We evaluated vaginal fibroblast proliferation, ECM deposition, and metabolism by quantification of collagen, elastin, and matrix metalloproteinases and by gene expression analysis for 28 days. We established effective E2 drug loading with a steady release over time. Significantly higher cell proliferation, collagen-, and elastin deposition were observed on electrospun P4HB scaffolds as compared to knitted P4HB. For this study, physical properties of the scaffolds were more determinant on the cell response than the release of E2. These results indicate that making these electrospun P4HB scaffolds E2-releasing appears to be technically feasible. In addition, electrospun P4HB scaffolds promote the cellular response of vaginal fibroblasts and further studies are merited to assess if their use results in improved surgical outcomes in case of POP repair.

## 1 Introduction

In women with pelvic organ prolapse (POP), there is a loss of supportive tissue strength and descent of the pelvic organs resulting in problems with micturition, defecation, and sexual functioning. It is a prevalent disorder affecting 40–50% of women, and the incidence increases with age.^1,2^ Women with POP have compromised vaginal fibroblast function and decreased extracellular-matrix (ECM) quality, thereby affecting the mechanical properties of the tissue.^3−5^ Despite the reduced tissue quality, native tissue repair (NTR) using patients’ own tissue is the first-line surgical treatment and is associated with a substantial risk of recurrence.^6−8^ To reduce this risk of recurrent prolapse, knitted polypropylene implants were introduced to provide durable mechanical support.^9^ However, while many patients have good long-term results, a considerable number of patients have suffered from serious implant-related adverse events such as mesh exposure or pain. This phenomenon is probably explained by a persistent inflammatory response toward permanent knitted implants.^10^

A potential solution might lie in a tissue-engineered approach using absorbable materials as these might elicit a milder inflammatory response as compared to permanent implants.^10^ In addition, as these implants are completely absorbed, certain complications can be resolved on its own over time. Besides, there has been a request by the Scientific Committee on Emerging and Newly Identified Health Risks (SCENIHR) of the European Union to study degradable implants.^11^ Our previous *in vitro* research on knitted delayed absorbable poly-4-hydroxybutyrate (P4HB) has shown promising results with respect to increased vaginal fibroblast proliferation and collagen deposition on P4HB as compared to knitted polypropylene implants.^12^*In vivo*, P4HB demonstrated a favorable host response with significantly higher M2/M1 ratios.^13^ We hypothesize that the cellular response to P4HB can be further improved by creating an electrospun P4HB scaffold. Electrospinning is a technique that uses an electric potential to create ultrathin polymeric fibers (micro- to nanoscale). Electrospun scaffolds have an adaptable large surface-to-volume ratio and a high porosity^14^ and thereby a more comparable structure with the natural ECM as compared to knitted implants. Consequently, electrospun scaffolds might favor cellular attachment, proliferation, and matrix production, favoring host tissue integration.^15^ In addition, electrospinning allows for the incorporation of certain antibiotics or hormones, creating implants that function as a (controlled) drug delivery system.^14^ As most POP patients are postmenopausal and in a hypo-estrogenic state, this may affect surgical outcomes as the sex hormone estrogen is of great importance for the wound healing capacity.^16^ Estrogen promotes vascularization, collagen synthesis, wound closure, and tissue strength and can decrease inflammation.^17^ By these effects, estrogen has beneficial effects on vaginal wound healing, possibly resulting in better pelvic floor function. Vaginal administration of estrogen has certain advantages compared to the oral route since there is no first-pass effect, it requires lower daily doses, and it provides continues release of medication.^18^ Consequently, we believe that the controlled release of estrogen at the surgical site can improve tissue regeneration and wound healing^19,20^ and that an estrogen-releasing implant benefits surgical outcomes.

Thus, the key is to develop a material that effectively mimics both the structure and function of the natural ECM and is biocompatible with the host tissue. For this study, we developed novel biodegradable estradiol (E2)-releasing electrospun P4HB scaffolds and described their characteristics. We aimed to study the *in vitro* cellular response of vaginal fibroblasts to electrospun P4HB scaffolds and assess outcomes relevant for POP repair. To test our hypothesis on the advantages of electrospun scaffolds over knitted implants, we compare our results to knitted P4HB. These steps might contribute to eventually finding a durable and safe surgical solution to treat women with POP.

## 2 Methods

### 2.1 Materials

#### 2.1.1 Materials and Spinning Details

Electrospun P4HB (ES P4HB) and 17β-estradiol releasing electrospun P4HB scaffolds (ES P4HB-E2) were fabricated by electrospinning (two high voltage supplies: ES60P-20W/CIC2 and ES30N-20W power unit (Gamma High Voltage Research, USA), rotating collector, and syringe pump (Chemyx Fusion 100, USA)). P4HB (8% w/w) was dissolved in CHCl_3_/DMF (9:1 w/w), and 17β-estradiol was added in increasing mass concentrations of 1%, 2%, and 5% (E2:P4HB w/w). Solutions were horizontally electrospun at 30% RH from syringes fitted with 19-G needles onto a rotating target (1000 rpm, Ø30 mm) at a collection distance of 300 mm with a flow rate of 4.2 mL/h using +15.4 kV (needle) and −4.4 kV (target) to achieve a scaffold thickness of 400 μm.

Knitted P4HB (Tepha, Inc., USA) with a diamond pore shape was selected as a comparison. Diamond has shown to have favorable characteristics, as it is least stiff and promotes fibroblast attachment, proliferation, and collagen deposition as compared to other P4HB implant designs.^12^ We have previously reported its detailed textural and mechanical characteristics.^12^ In summary, the knitted implant is 0.28 mm thick, with a fiber diameter of 100 μm and pore size of 2.22 mm^2^.

#### 2.1.2 Characteristics

Electrospun scaffolds were imaged using scanning electron microscopy (SEM) (Phenom ProX Desktop SEM, Thermo Fisher, USA) after gold sputter coating (Polaron SC7640, Quorum Technologies, UK). The average fiber diameters and pore sizes were calculated using ImageJ (v1.52q, NIH Image, USA). The porosity of the scaffolds was measured using the differential mass of the scaffold in air versus the scaffold mass in a heptane solution and calculated using the following equation:
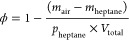
where *m*_air_ and *m*_heptane_ are the masses of the samples in air and suspended in heptane, respectively, ρ_heptane_ is the density of heptane, and *V*_total_ is the bulk density of the porous sample.

#### 2.1.3 Estradiol Release

To assess *in vitro* E2 release, 10 mm^2^ electrospun samples were weighed and incubated in sealed tubes containing 1 mL of PBS (pH 7.4) at 37 °C. E2 release was assessed for 28 days, using UV spectrometry (Shimadzu UV-1601PC, Japan), at a wavelength of 200 nm. The concentrations were calculated using a standard absorbance curve of known E2 concentrations.

### 2.2 Cell–Matrix Interactions

#### 2.2.1 Cell Seeding

Primary vaginal fibroblasts were isolated from a postmenopausal patient undergoing anterior wall prolapse surgery as previously described.^21^ Postmenopausal cells were chosen as postmenopausal patients are the main target group for these E2-releasing scaffolds. Scaffolds were cut in 10 × 10 mm^2^ samples under sterile conditions and transferred to flat 24-well plates. Vaginal fibroblasts at passage 3 or 4 were seeded on day 0 onto the scaffolds at a density of 20 000 cells/cm^2^ in 1 mL of Dulbecco’s modified Eagle’s medium/Nutrient Mixture F-12, no phenol red (DMEM/F-12) (Gibco-Life technologies, UK) supplemented with 10% v/v fetal bovine serum (FBS) (Gibco-Life technologies, UK), and 2% Penicillin–Streptomycin (10 000 U/mL) (Gibco-Life technologies, UK). The scaffolds were incubated for a total of 28 days at 37 °C with 5% CO_2_ in a humidified environment and the medium was refreshed every 3–4 days. Culture medium without phenol red was chosen, as phenol red can have a weak estrogenic activity that could interfere with the influence of the estrogen functionalized scaffolds on our postmenopausal cells.^22^

#### 2.2.2 Cell Proliferation

At days 1, 7, 14, 21, and 28, vaginal fibroblast proliferation on scaffolds was assessed by a continuous Alamar blue colorimetric viability assay (Bio-Rad Laboratories, Inc. USA) based on metabolically active cells reducing resazurin (600 nm) to resorufin (570 nm). Three independent experiments were performed and per experiment three samples per scaffold type were evaluated. The scaffolds were transferred to a clean 24-well plate and incubated with 600 μL of 10% Alamar Blue (10% v/v Alamar blue in supplemented DMEM) at 37 °C with 5% CO_2_. After 3 h, the optical density (OD) in 150 μL of the Alamar blue solution was read trifold in a flat-bottom 96-well plate at 570 and 600 nm (Synergy H1 multimode microplate reader, Biotek Instruments Inc. USA). The average OD of three scaffolds was calculated after correction with the OD of a cell-free control mesh and the culture medium.

#### 2.2.3 Scanning Electron Microscopy (SEM) for Fibroblast Distribution

Cell proliferation and ECM deposition on the scaffolds were imaged after 14 and 28 days of incubation using SEM. The samples were fixed in 4% paraformaldehyde and 1% glutaraldehyde for 4 h at room temperature, followed by dehydration using a graded ethanol series. The samples were immersed in hexamethyldisilizane (Sigma-Aldrich, USA) for 30 min to reduce sample surface tension. After air-drying, the samples were mounted on aluminum stubs and sputter-coated with a 6 nm platinum–palladium layer using a Leica EM ACE600 sputter coater (Leica Microsystems, Germany). Micrographs were taken at random locations at a magnification of 100× using a Zeiss Sigma 300 scanning electron microscope (Zeiss, Germany).

#### 2.2.4 Cytoskeleton Morphology

After a 15 min fixation in 4% paraformaldehyde on days 14 and 28, and permeabilization of the cells with 0.1% Triton X-100, the scaffolds were stained for fluorescence imaging for 30 min at room temperature with Alexa Fluor 488 Phalloidin (Invitrogen, Carlsbad, USA) staining the F-actin of the cytoskeleton, and DAPI (4′,6-diamidino-2-phenylindole dihydrochloride) (Sigma-Aldrich, USA) as a nuclear counterstain for 3 min. The scaffolds were imaged using a fluorescent microscope (Leica DM5000 B, Leica, Germany).

#### 2.2.5 Collagen Deposition

Collagen deposition was evaluated on days 14 and 28 using 0.1% Picrosirius Red (PSR) staining. After fixation as described above, samples were stained for 30 min in 400 μL PSR at room temperature. The stained scaffolds were imaged using a light microscope (Olympus BX41, Leica, Germany). In addition, collagen deposition was semiquantitatively assessed by measuring the absorbance of the extracted dye at 540 nm. For this, after staining, the samples were washed three times with 1 mL of PBS, and 1 mL of extraction buffer (Chrondrex, Inc., USA) was added. After resuspension, the absorbance was read trifold in 150 μL in a flat-bottom 96-well plate at 540 nm (Synergy H1 multimode microplate reader, Biotek Instruments Inc. USA). Using standard reference curves based on rat collagen I (Cultrex, R&D systems, USA), the amount of collagen was calculated in μg/scaffold. Three independent experiments were performed, and per experiment three samples per scaffold type were evaluated.

#### 2.2.6 Elastin Content

Elastin content was evaluated on days 14 and 28 using the Fastin elastin assay kit (Biocolor, UK) following the manufacturer’s protocol. Two independent experiments were performed, and per experiment two samples per scaffold type were evaluated. In short, after detachment of the cells from the scaffolds, cell-bound elastin was converted to α-elastin by the addition of 1.0 M oxalic acid and heating to 100 °C for 1 h. After elastin precipitation, the dye was added and samples were incubated for 90 min. This was followed by adding a dye dissociation reagent and the reading of the OD at 513 nm in a 96-well plate (Synergy H1 multimode microplate reader, Biotek Instruments Inc. USA).

#### 2.2.7 Matrix Metalloproteinase Activity

The enzymatic activity of matrix metalloproteinases (MMPs) in cell culture supernatants was assessed using gelatin zymography by the gelatinolytic activity of MMP-2 and MMP-9.^23^ Two independent experiments were performed, and per experiment three samples per scaffold type were evaluated. In short, culture media were replaced by culture media containing 1% FBS for 24 h before the conditioned media were obtained. The supernatant was taken, and the Pierce BCA Protein Assay (Thermo Scientific, USA) was performed to determine the total protein content in each sample following the manufacturer’s protocol. Wells of self-casted zymography gels containing 1% gelatin were then loaded with diluted samples mixed with sample buffer, each containing 5 μg of total protein.^24^ One well per gel was loaded with Precision Plus Protein Dual Color Standard (Bio-Rad, USA) as a reference for the identification of MMPs. Gel electrophoresis was applied to separate MMPs depending on molecule size with MMP-2 between 50 and 75 kDa and MMP-9 between 75 and 100 kDa. After electrophoresis, gels were placed into renaturing buffer and hereafter in the developing buffer overnight. The gel was stained using SimplyBlue and destained with deionized water. The intensity of the digested gelatin bands was measured using ImageJ (1.50i, NIH, USA) by calculating the densitometry peak area (DPA). The data were normalized per μg of protein to correct for differences in cell number per experiment.

#### 2.2.8 Gene Expression for ECM Components and Tissue Remodeling

Gene expression for collagen I, III, elastin, MMP-2, MMP-9, and α-SMA was analyzed by q-PCR on day 28. Cells were detached from the scaffolds by trypsin-EDTA (0.25%) (Gibco-Life technologies, UK) and the cells from 5 scaffolds were collected in one tube. Cells were lysed in a 1% β-mercapthoethanol in RLT buffer (Qiagen, Germany) and stored at −80 °C. RNA was isolated using RNeasy Mini Kit (Qiagen, Germany) according to the manufacturer’s protocol. RNA concentration and purity were determined using Nanodrop 1000 (Thermo Fisher, USA) and diluted to obtain a concentration of 200 ng of RNA in sterile water. cDNA was synthesized by adding a reversed transcriptase mix of random primers (Roche, Switzerland) and Superscript II Reverse Transcriptase (Thermo Fisher, USA). qPCR was performed by adding SYBR Green I Mastermix to cDNA and measuring Cp-values using the Lightcycler 480 (Roche, Switzerland). Gene expression was presented relative to housekeeping genes HPRT-1 and YWHAZ after calculation using the delta–delta Ct method.^25^ Primer sequences for the target genes are listed in Table 1. Two independent experiments were performed and, in each, three samples per scaffold type were evaluated.

**Table 1:** Primer Sequences^a^

target gene	forward primer	reverse primer
COL1A1	TCCAACGAGATCGAGATCC	AAGCCGAATTCCTGGTCT
COL3A1	GATCCGTTCTCTGCGATGAC	AGTTCTGAGGACCAGTAGGG
ELN	TTTGGCCCGGGAGTAGTTGG	CAGCTGCTTCTGGTGACACAAC
MMP2	TCCAAGTCTGGAGCGATGTG	CCGTCCTTACCGTCAAAGGG
MMP9	GAGGTGGACCGGATGTTCC	AACTCACGCGCCAGTAGAAG
ACTA2	CCTGACTGAGCGTGGCTATT	GATGAAGGATGGCTGGAACA
HPRT1	CTCAACTTTAACTGGAAAGAATGTC	TCCTTTTCACCAGCAAGCT
YWHAZ	GATGAAGCCATTGCTGAACTTG	CTATTTGTGGGACAGCATGGA

a Primer sequences for the target genes of collagen I and III, elastin, MMP-2, and α-SMA. Primer sequences for housekeeping genes HPRT1 and Ywhaz. COL1A1, α1(I)procollagen; COL3A1, α1(III)procollagen; ELN, elastin; HPRT, hypoxanthine phoshoribosyltransferase; YWHAZ, tyrosine 3-monooxygenase/tryptophan 5-monooxygenase activation protein, zeta polypeptide.

### 2.3 Statistical Analysis

Analyses were performed in GraphPad Prism version 9.1.0 for Windows (GraphPad Software, USA). Scaffold characteristics were reported as mean ± standard deviation (SD), and groups were compared using a one-way ANOVA. Since other data were not normally distributed, results were expressed as median and interquartile range (IQR), and the different scaffolds were compared using a Mann–Whitney U test or a Kruskal–Wallis test. A *p*-value of <0.05 was considered statistically significantly different. Correction for multiple testing was performed for the outcomes of the proliferation assay considering the number of comparisons within this experiment by a Bonferroni correction (compare different time points) or a Dunn’s multiple comparisons test (compare scaffolds within a time point).

## 3 Results

### 3.1 Scaffold Characteristics and Estradiol Release

**Figure 1:**
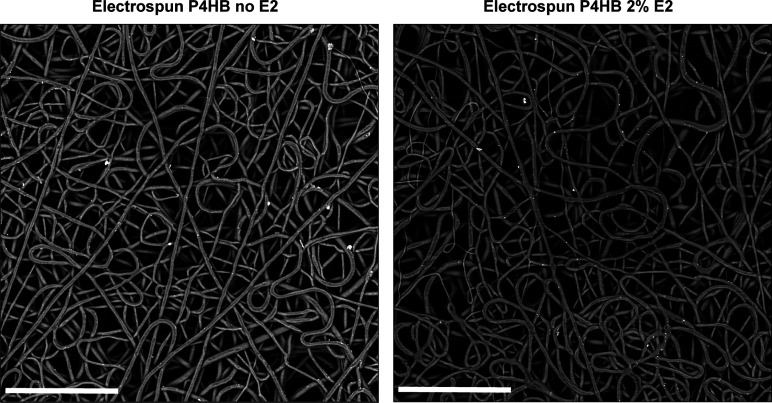
Scaffold architecture. Scanning electron microscopy images of electrospun scaffolds containing no and 2% E2, respectively (as indicated), each at 1000× original magnification (scale bars represent 80 μm).

Electrospun P4HB scaffolds demonstrated effective E2 drug incorporation, with no crystals seen on the fiber surfaces (Figure 1). The average diameter of ES P4HB scaffolds was 3.41 ± 0.57 μm, and although there was a statistical difference compared to E2 containing groups regardless of concentration (all *p* < 0.001), the difference between the largest and smallest group was small (0.34 μm) (Table 2). The pore diameters were statistically indistinguishable, with pores ranging from 13.51 ± 1.30 μm to 15.36 ± 0.92 μm (not significant (NS)). The addition of E2 did have a significant but small (less than 3%) effect on porosity, with a decrease from 76.14 ± 0.67% to 73.26 ± 0.78% with the inclusion of 5% E2 (*p* = 0.008). Other groups did not demonstrate significant differences.

**Table 2:** Morphological Characteristics^a^

	P4HB no E2	P4HB 1% E2	P4HB 2% E2	P4HB 5% E2
fiber diameter (μm)	3.41 (0.57)	3.14 (0.54)	3.16 (0.58)	3.07 (0.46)
pore size (μm^2^)	13.51 (1.30)	15.36 (0.92)	15.34 (1.21)	14.47 (0.80)
porosity (%)	76.14 (0.67)	74.69 (0.97)	75.08 (0.62)	73.26 (0.78)

a Fiber diameters, pore sizes (reported as equivalent pore diameter), and porosities for electrospun scaffolds containing no, 1%, 2%, and 5% estradiol (E2). Results expressed as mean (standard deviation).

*In vitro* E2 release was compared for the three E2-containing electrospun scaffolds. A cumulative release of 6.60 μg, 5.77 μg, and 4.69 μg of E2 was seen in the first 7 days from the ES P4HB-E2 1%, 2%, and 5% E2 groups, respectively. This increased to 12.98 μg, 11.57 μg, and 10.96 μg by day 28 for the three respective increasing concentrations, which equated to 1.71%, 1.18%, and 0.12% of total drug in the scaffolds, respectively, released in the first 7 days, and 14.85%, 7.60%, and 2.04% of the drug total content by day 28. At the end point of the study, there was still E2 being released. When the cumulative release in μg/mg E2 was compared, the difference was statistically significant for all E2-containing scaffolds from day 7 on.

### 3.2 Cell–Matrix Interactions

#### 3.2.1 Cell Proliferation and Matrix Morphology

**Figure 2:**
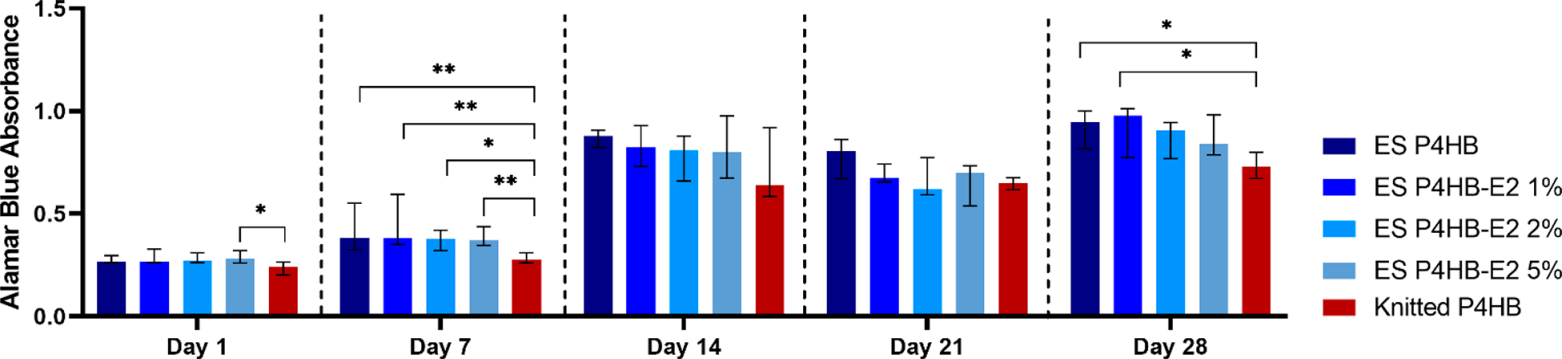
Cell proliferation. Cell proliferation was measured using an Alamar blue colorimetric viability assay on days 1, 7, 14, 21, and 28. Cell proliferation was significantly higher for certain electrospun (ES) P4HB scaffolds as compared to knitted P4HB on days 1, 7, and 28. In addition, cell proliferation increased significantly over time (day 1 vs day 7: all ES P4HB, day 7 vs day 14: all scaffolds, day 21 vs day 28: ES P4HB no E2, ES P4HB-E2 1%, 2%, and 5%), not shown in figure. Data are reported as median with IQR. E2, estradiol. *p < 0.05, **p < 0.01.

Cell proliferation increased over time on both the knitted and electrospun P4HB scaffolds (Figure 2). In the first 7 days, there was already a significant increase for all electrospun scaffolds (all *p* < 0.001) when comparing cell proliferation at days 1 and 7, but not for knitted P4HB. The most pronounced increase in proliferation was seen between days 7 and 14 for all scaffolds (all *p* < 0.001). Overall, cell proliferation on electrospun P4HB scaffolds with and without E2 was higher as compared to knitted P4HB on all time points, which reached a statistically significant difference at day 1 (knitted vs ES P4HB-E2 5% (*p* = 0.04)), day 7 (knitted vs ES P4HB (*p* = 0.007), vs ES P4HB-E2 1% (*p* = 0.002), vs ES P4HB-E2 2% (*p* = 0.03) and vs ES P4HB-E2 5% (*p* = 0.009)), and on day 28 (knitted vs ES P4HB (*p* = 0.02) and vs ES P4HB-E2 1% (*p* = 0.03)). There were no significant differences between ES P4HB and ES P4HB-E2.

**Figure 3:**
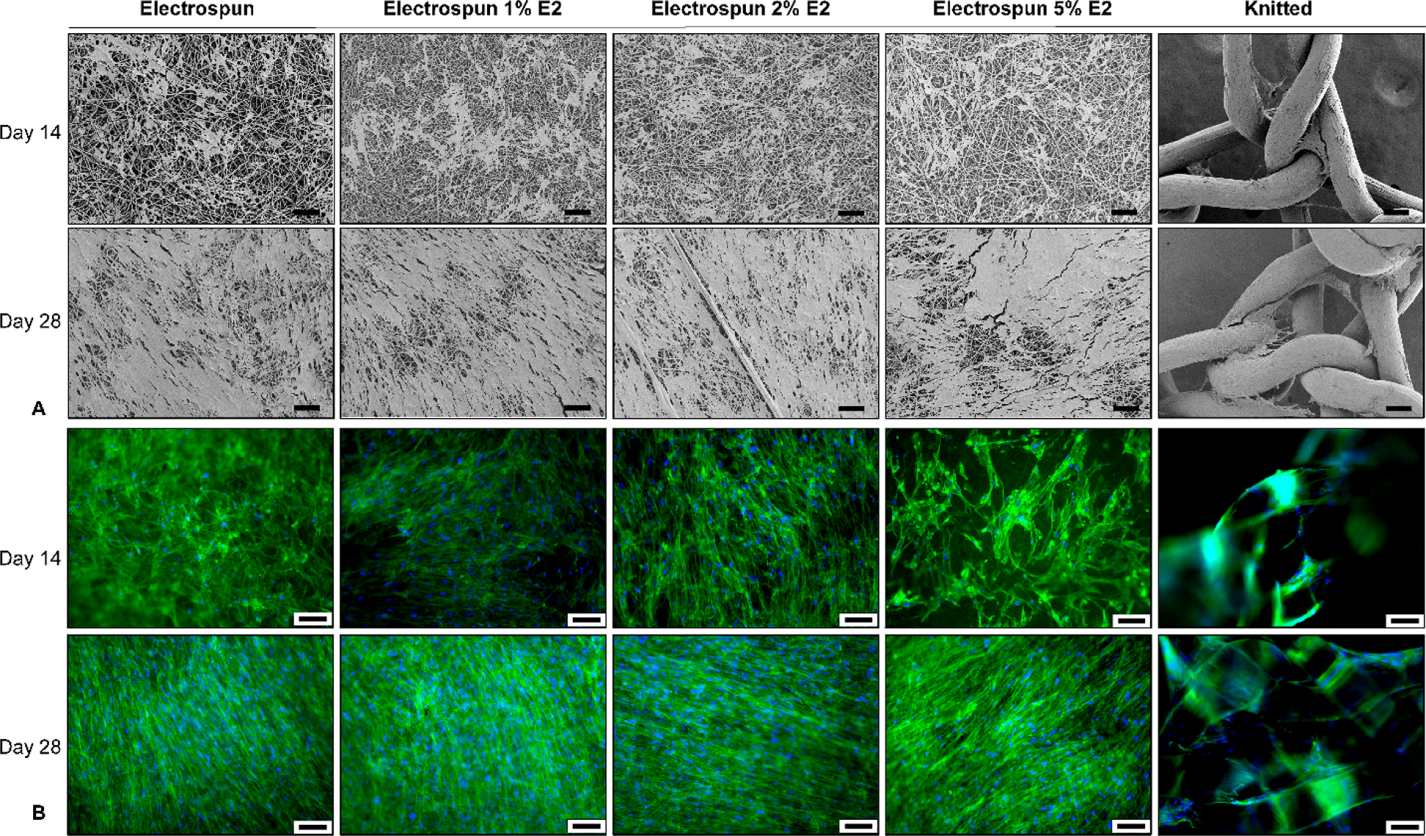
Vaginal fibroblast proliferation, extracellular matrix (ECM) deposition, and cytoskeleton morphology. Vaginal fibroblast and ECM deposition were imaged using (A) scanning electron microscopy and (B) cytoskeleton morphology using fluorescent imaging of the cytoskeleton (green) and nuclei (blue) on day 14 and day 28. These representative images demonstrated an ECM increase over time and more evenly distributed on the electrospun scaffolds. The different scale bars all represent 100 μm.

Imaging of the ECM and cytoskeleton (using SEM and fluorescent imaging) demonstrated that vaginal fibroblasts attached, proliferated, aligned, and spread on all electrospun P4HB scaffolds (Figure 3). At day 14, ECM deposition could be seen, and at day 28, the ECM covered the surfaces of the scaffolds and an increase in cell alignment could be visualized. In addition, cell attachment and ECM distribution were more confluent and even over the electrospun P4HB scaffolds when compared to the knitted P4HB, on which only the fibers and knots were covered. At day 14, F-actin stress fibers of the fibroblasts attached to the knitted P4HB, ES P4HB, and ES P4HB-E2 1% and 2% were aligned in the long axis of the cells. The fibroblasts on ES P4HB-E2 5% exhibited more and randomly oriented stress fibers than the other scaffolds at day 14. However, at day 28, stress fibers of the fibroblasts on the ES P4HB and ES P4HB-E2 were extended and cells were distributed on the electrospun scaffold surface showing more cellular interconnections as compared to knitted P4HB.

#### 3.2.2 Collagen Deposition

**Figure 4:**
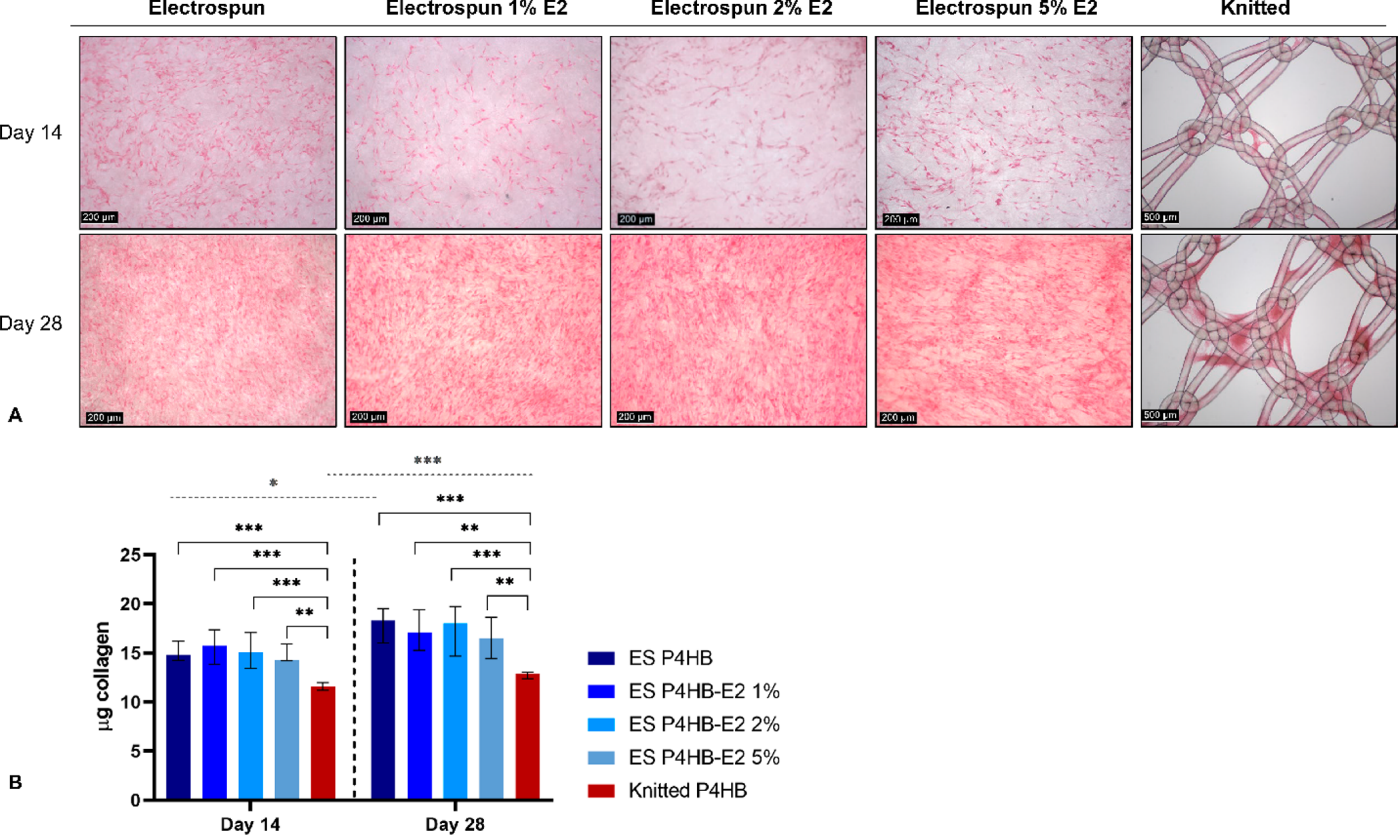
Collagen deposition. Collagen deposition was imaged after a Picrosirius Red stain on days 14 and 28. (A) Representative images demonstrate increased collagen deposition over time and collagen was more evenly distributed over the electrospun (ES) P4HB scaffolds as compared to the knitted P4HB. (B) Collagen deposition increased over time, which reached significant differences for ES P4HB and knitted P4HB (gray dashed line above figure). Collagen deposition was significantly higher for all ES P4HB as compared to knitted P4HB on both days 14 and 28 (black brackets). Data is reported as median with IQR. *p < 0.05, **p < 0.01, ***p < 0.001.

Picrosirius Red staining confirmed that collagen deposition on the knitted P4HB was mostly seen around the knots, whereas on all electrospun scaffolds, collagen was more evenly distributed (Figure 4A). An increase in collagen deposition could be seen on all scaffolds between day 14 and day 28 (Figure 4A). However, semiquantitative readings of the absorbance revealed only a significant increase on ES P4HB (*p* = 0.02) and knitted P4HB (*p* < 0.001), as collagen deposition was already high on other electrospun scaffolds at day 14 (Figure 4B). Moreover, collagen deposition was significantly more on all electrospun scaffolds as compared to the knitted P4HB on both day 14 (knitted vs ES P4HB (*p* < 0.001), vs ES P4HB-E2 1% (*p* < 0.001), vs ES P4HB-E2 2% (*p* < 0.001), and vs ES P4HB-E2 5% (*p* = 0.001)) and day 28 (knitted vs ES P4HB (*p* < 0.001), vs ES P4HB-E2 1% (*p* = 0.001), vs ES P4HB-E2 2% (*p* < 0.001), and vs ES P4HB-E2 5% (E2 *p* = 0.003)).

#### 3.2.3 Elastin Deposition

**Figure 5:**
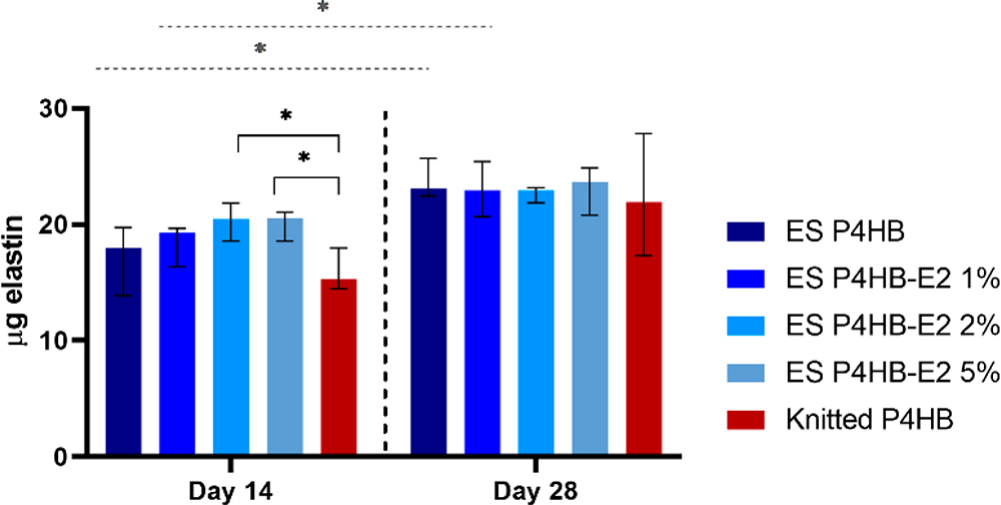
Elastin content. Elastin content was measured using a Fastin elastin assay on days 14 and 28. The elastin content increased significantly over time for electrospun (ES) P4HB and ES P4HB-E2 1%. Elastin content was significantly higher for ES P4HB-E2 2% and 5% compared to knitted P4HB. Data are reported as median with IQR. *p < 0.05

Elastin content increased over time and was higher for the electrospun P4HB scaffolds as compared to the knitted P4HB (Figure 5). Furthermore, the addition of E2, and higher concentrations of E2, resulted in an increase of the elastin content especially at the early time point reaching significant differences for knitted vs ES P4HB-E2 2% (*p* = 0.02) and vs ES P4HB-E2 5% (*p* = 0.03).

#### 3.2.4 MMP-2 Activity

**Figure 6:**
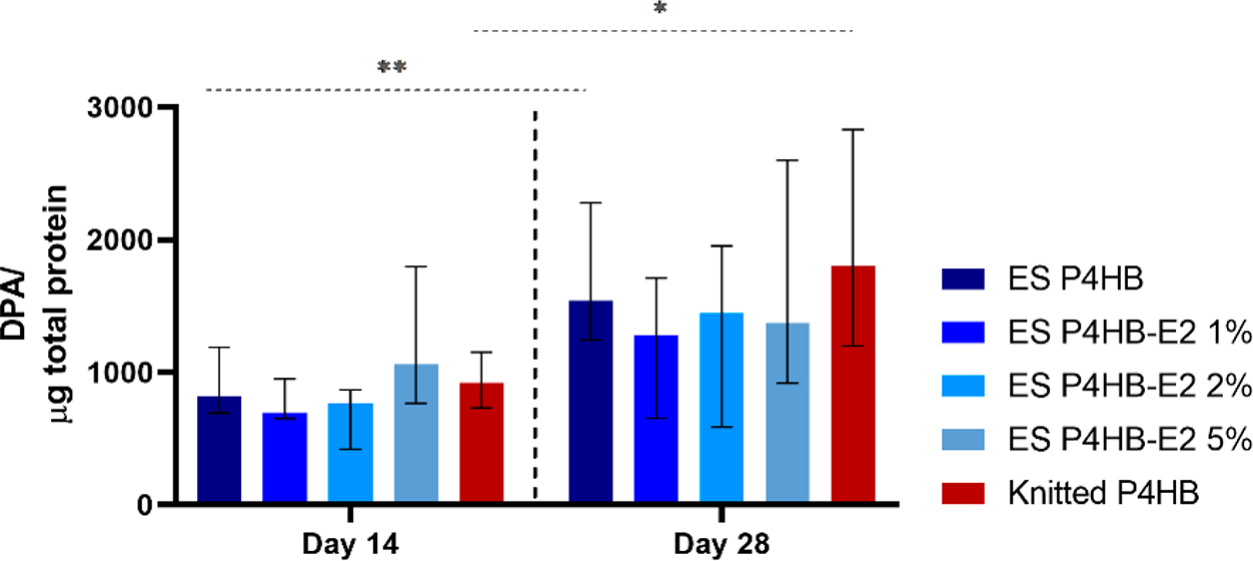
MMP-2 activity. Pro-active MMP-2 activity on days 14 and 28. The MMP-2 increased significantly over time for electrospun (ES) P4HB and knitted P4HB. No significant differences could be observed between knitted and ES P4HB. Data are reported as median with IQR. *p < 0.05, **p < 0.01.

Only pro-active MMP-2 was found in our samples, and it increased over time (Figure 6), reaching significant differences for ES-P4HB and knitted P4HB. At day 28, pro-active MMP-2 levels from the knitted P4HB were higher than those of all the electrospun scaffolds, though this did not reach statistically significant differences when all scaffolds were compared.

#### 3.2.5 Gene Expression

**Figure 7:**
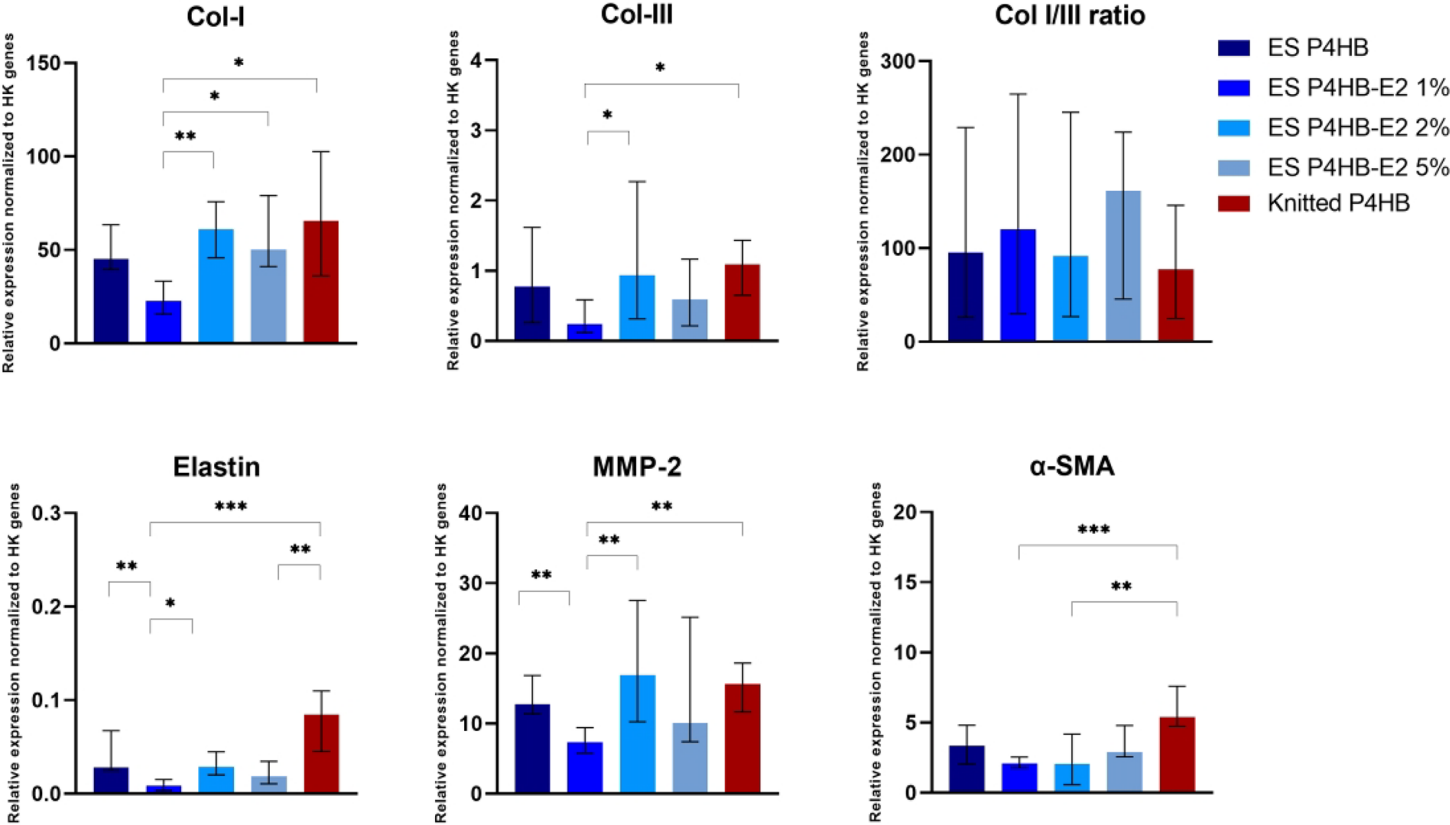
q-PCR. This figure demonstrates the relative expression of the target genes collagen I and III, elastin, MMP-2, and α-SMA normalized to housekeeping (HK) genes HPRT-1 and Ywhaz at day 28. Data are reported as median with IQR. *p < 0.05, **p < 0.01, ***p < 0.001.

We found at day 28 significantly higher collagen-I gene expression by fibroblasts on ES P4HB-E2 2% and 5% and knitted P4HB as compared to ES P4HB-E2 1% (*p* = 0.003, *p* = 0.02, and *p* = 0.01, respectively) and higher collagen-III gene expression in ES P4HB-E2 2% and knitted P4HB as compared to ES P4HB-E2 1% (*p* = 0.03 and *p* = 0.02, respectively) (Figure 7). Elastin gene expression was significantly higher in knitted P4HB as compared to ES P4HB-E2 1% (*p* < 0.001) and ES P4HB-E2 5% (*p* = 0.003), and in ES P4HB and ES P4HB-E2 2% as compared to ES P4HB-E2 1% (*p* = 0.009 and *p* = 0.03, respectively). In addition, MMP-2 gene expression was significantly higher in ES P4HB, ES P4HB-E2 2%, and knitted P4HB as compared to ES P4HB-E2 1% (*p* = 0.008, *p* = 0.004, and *p* = 0.003). Finally, α-SMA gene expression was significantly higher in knitted P4HB as compared to ES P4HB-E2 1% (*p* < 0.001) and 2% (*p* = 0.005).

## 4 Discussion

By assessing vaginal fibroblast attachment, proliferation, and functioning, we found certain favorable effects of electrospun P4HB scaffolds. Though not all outcomes demonstrated significant differences in this *in vitro* study, electrospun P4HB demonstrated at certain time points increased cell proliferation, collagen and elastin deposition, and ECM formation as compared to knitted P4HB. In addition, effective E2 drug loading was established with a steady release over time, ongoing after the 28 days duration of our study.

Pelvic floor disorders are a common problem, and surgical interventions can improve women’s quality of life.^26^ However, there is no optimal first line treatment, and there has been a growing interest in pelvic floor tissue engineering. For the current study, we produced our own absorbable electrospun P4HB scaffolds. P4HB was chosen as knitted P4HB implants have shown promise in reconstructive surgery for ventral hernia repair^27^ and in our preclinical studies for pelvic floor repair.^12,13,28,29^ In addition, electrospun P4HB for other applications such as a dural^30^ or dermal^31,32^ substitution are encouraging, but the available studies are limited, and no studies on electrospun P4HB for pelvic floor repair have been conducted. Because fibroblasts are the main cell type of the connective tissue of the vaginal wall and are responsible for maintaining the ECM,^3,33^ their function is critical in assessing implant performance in the pelvic floor and to predict how the implant will interact with the host. Consequently, this study contributes in collecting the evidence required in the preclinical study phase of new implants for pelvic floor repair.^34^

Our results demonstrated faster and increased vaginal fibroblast proliferation and ECM production on all electrospun P4HB scaffolds as compared to knitted P4HB. Already within the first week, we observed a significant increase in cell proliferation on electrospun P4HB. An explanation lies in the different microstructure of the electrospun scaffolds, as their higher surface area and porosity could result in better cell adhesion and functioning.^35^ The higher porosity of scaffolds provides more attachment points to cells, meaning that the implant’s microstructure is conducive to cell seeding allowing cellular ingrowth.^15^ Scaffold pore size is vitally important since it dictates whether cells view the scaffold as two- or three-dimensional.^14^ Our pore sizes were found to be above 8 μm, which is required for cellular ingrowth.^36^ Moreover, porosity was above 70% allowing cells to migrate through the scaffold as well as accommodate for space needed for nutrient and waste transportation.^37^ Partially as a consequence of increased proliferation, deposition of collagen, elastin, and ECM were higher on electrospun scaffolds compared to knitted P4HB. Besides, while on knitted P4HB cells adhered mostly to the knots, on electrospun P4HB, cells spread over the entire surface of the scaffold and more F-actin stress fibers were observed. This could allow cells on the scaffolds to maintain tension and adapt to mechanical stress. To maintain the ECM integrity, there should be a fine balance between the synthesis of collagen and elastin and its degradation. Enzymes regulating ECM tissue remodeling and responsible for matrix degradation are MMPs. It has been demonstrated that especially MMP-2 and MMP-9 are upregulated in the vaginal wall of women with POP, resulting in increased collagen turnover.^3^ Zymography showed that active MMP-2 and MMP-9 were below detection, consistent with findings of others.^21,38,39^ Also with gene expression analysis, MMP-9 was not detected. However, pro-active MMP-2 levels (zymography) and MMP-2 (q-PCR gene analysis) at day 28 were relatively high in cell cultures on knitted P4HB as compared to electrospun P4HB. *In vivo*, this could lead to defective and mechanically weaker newly formed tissue as increased levels of MMP-2 are associated with structurally compromised tissue^40^ due to increased collagen and elastin degradation.^41^

As said, at the protein level, electrospun P4HB exhibited higher cell proliferation, collagen, elastin, and ECM deposition. In addition, gene expression analyses also support the idea of a delayed cellular response to knitted P4HB as compared to electrospun ones. We found that collagen-I gene expression was higher for all electrospun scaffolds than the expression of collagen-III, and even though the differences were not statistically significant, the collagen-I/III ratio was higher for the electrospun scaffolds. In *in vivo* tissue remodeling, immature collagen-III is replaced by mature collagen-I, and elastin deposition in the tissue increases over time. Increasing collagen-I/III ratio contributes relevantly to the structural integrity of supportive tissue in the pelvic floor and an increased ratio could lead to enhanced remodeling, more optimal wound healing, and improved tissue strength.^5,33,42^ In addition, elastin and α-SMA gene expression were significantly higher at day 28 in knitted as compared to certain electrospun P4HB constructs, which suggests relatively slow ECM synthesis and remodeling. α-SMA is a marker for fibroblast–myofibroblast transition and a precursor for collagen synthesis.^5^ Myofibroblasts can cause tissue contraction with the help of their cytoplasmic microfilaments (actin-rich stress fibers).^5^ Prolonged or excessive myofibroblastic differentiation can result in detrimental tissue fibrosis,^43,44^ which was also seen clinically with former knitted polypropylene implants and can cause certain complications like pain.

Adding estrogen to the surgical site could promote *in vivo* pelvic floor tissue repair by promoting wound healing. Estrogen has an effect on the inflammatory response by the release of pro- and anti-inflammatory cytokines, it stimulates neovascularization, and estrogen promotes fibroblast proliferation and collagen and ECM synthesis.^17,19,20^ In this study, we demonstrate the successful incorporation of E2 into the fibers of an electrospun P4HB scaffold that did not substantially alter the overall microstructure of the mesh. These findings were consistent with those of MacNeil et al.^45,46^ who also demonstrated E2 incorporation into the electrospun polyurethane (PU) and poly(l)-lactic acid (PLA) fibers, without affecting the microstructure of the polymer scaffolds produced. The effective incorporation can be ascribed to the lipophobic nature of E2 and its high solubility in DMF. Over a period of 28 days, we achieved a sustained release with evidence of E2 still being released after this period. After an initial burst release during the first 7 days, E2 was released in a linear fashion from day 10 until the rest of the 28 day study period. Yet, despite this burst, the P4HB scaffolds only experienced a release of 10% of the loaded drug in the first 10 days, compared to others who experienced a burst release of about 30–40% within the first 10 days when E2 was incorporated into PU^45^ or 40–50% by day 14 when incorporated in PLA.^46^ Our confined burst release demonstrates adequate drug integration, scaffold stability, and ensures a sustained release over time. Recently, a drug-eluting 3D printed and coaxial electrospun polycaprolactone (PCL) poly(lactic-co-glycolic acid) (PLGA) scaffold has demonstrated rather comparable E2 releasing profiles; however, this scaffold also released lidocaine and metronidzole.^47^

In this *in vitro* study, we only assessed the effect on a cellular level and we observed that E2 seemed to promote early elastin deposition as there was a trend toward increased elastin at day 14 with increasing E2 concentrations. Moreover, there was an increased gene expression of collagen-I, collagen-III, elastin, and MMP-2 for ES P4HB-E2 2% as compared to other electrospun scaffolds. This may imply that the vaginal fibroblasts were stimulated to synthesize factors involved in ECM deposition and remodeling at 2% E2 concentration. It was found previously that fibroblast activity may be promoted at a certain level of exposure to E2 rather than showing a linearly increasing dose response.^48^ Even though the addition of E2 to electrospun P4HB scaffolds had not been reflected in enhanced cellular functions at the protein level, gene-level data could possibly mean *in vivo* enhanced tissue regeneration, healing, and increased mechanical strength with certain E2 concentrations. *In vitro*, E2 incorporation in other electrospun scaffolds has demonstrated to increase collagen and elastin production.^45,46^ Although many beneficial effects of E2 have been reported, some studies suggest an unfavorable effect of E2 on *in vitro* POP fibroblast proliferation^49^ and vaginal smooths muscle cells elastin deposition.^50^ From our results, we can conclude that E2 did not cause any unfavorable effects.

While various polymers have been successfully electrospun in the past years for pelvic floor repair, *e.g*., PLA,^51^ PCL,^52−55^ poly(l-lactide-co-caprolactone) (PLCL),^56^ poly(lactic-co-glycolic acid)-blended-poly(caprolactone) (PLGA/PCL),^36,57^ PU,^45,58^ ureidopyrimidinone-polycarbonate (UPy-PC),^58^ or nylon,^57^ none has found widespread clinical introduction as disappointing preclinical results can prevent use in human studies. For example, while short-term follow-up studies on PCL demonstrated promising results with respect to biomechanical properties,^52^ PCL is on the long-term associated with inflammation, implant encapsulation, poor tissue integration,^54^ and clinical failure.^53,55^ And while the inflammatory response to UPy-PC is mild, and no implant-related complications have been reported during 180 days of follow-up, concerns have been raised about the fast degradation in case of clinical application.^58^ These examples indicate that preclinical studies are required to assess scaffold potential, and previously studied electrospun scaffolds have their own limitations so there is an ongoing search for a new material. We are the first to produce and investigate a pelvic floor implant made of electrospun P4HB, which can function as a drug delivery scaffold releasing E2. While previous studies have demonstrated favorable effects of electrospun as compared to knitted scaffolds, these scaffolds are often made of different polymers. For example, when compared to a knitted polypropylene implant, electrospun PLA has demonstrated increased proliferation.^51^ This makes it hard to attribute certain effects, and for this reason, we compared our results to knitted P4HB. A limitation of this study is that these results were obtained in a controlled *in vitro* experimental setup and we do not know which exact E2 concentrations *in vivo* would be ideal. In addition, due to the limited number of samples, we could not include earlier time points for gene expression analysis. Finally, we used cells isolated from one postmenopausal POP patient, and it should be noted that there might be variations in cell behavior between individuals. However, by using diseased cells from a postmenopausal POP patient, it helps us to better mimic the *in vivo* situation as POP fibroblasts differ from healthy fibroblasts at morphological, enzymatic, and gene expression levels.^21,38^

So, the behavior of the cells on a scaffold surface in terms of attachment and spreading eventually influences cell behavior such as cellular growth and intercellular interactions among each other, thereby their function due to enhanced cellular signaling.^12,59^ In our case, increased cell proliferation, and thereby increased ECM deposition on electrospun scaffolds as compared to knitted P4HB, might be a result of better functioning of cells on the ECM-mimicking structure of electrospun scaffold. As the mechanical properties of the vaginal wall depend on collagen and elastin and deficiencies are associated with the development of POP,^3^ these results *in vivo* would most likely result in increased tissue strength and are thereby essential for successful outcomes in the long term. Ultimately, by the implantation of a biodegradable scaffold *in vivo*, an appropriate inflammatory response might be induced leading to ECM synthesis and remodeling and establishing sufficient tissue strength by the time the implant is degraded.^60^

## 5 Conclusion

To our knowledge, this is the first study investigating the potential of P4HB scaffolds with controlled release of E2 for the treatment of POP. Electrospun P4HB scaffolds appear to promote the cellular response of vaginal fibroblasts in comparison to knitted P4HB, as indicated by increased cell proliferation and matrix deposition on electrospun scaffolds. This difference might be explained by the ECM-like microstructure of electrospun scaffolds, which provides more surface for early fibroblast attachment, survival, and fibroblast proliferation. An additional advantage of electrospinning is the opportunity to create a P4HB drug delivery system. We found effective incorporation of E2 and steady and prolonged release up until at least 4 weeks. However, on a cellular level, the microenvironment that the scaffold structure creates is influencing vaginal fibroblast functioning more than the addition of E2 in this *in vitro* study. As a next step, *in vivo* studies evaluating the host response and effects of mechanical loading should provide more insight into the potential beneficial effects of novel absorbable E2-releasing electrospun P4HB scaffolds.

## Notes

The data that support the findings of this study are available from the corresponding author, upon reasonable request.
